# Effects of robot-assisted gait training within 1 week after stroke onset on degree of gait independence in individuals with hemiparesis: a propensity score-matched analysis in a single-center cohort study

**DOI:** 10.1186/s12984-025-01581-4

**Published:** 2025-02-28

**Authors:** Daisuke Kato, Satoshi Hirano, Daisuke Imoto, Takuma Ii, Takuma Ishihara, Daisuke Matsuura, Hirofumi Maeda, Yoshitaka Wada, Yohei Otaka

**Affiliations:** 1 Department of Rehabilitation, Fujita Health University Hospital, 1-98 Dengakugakubo, Kutsukake-cho, Toyoake, 470-1192 Aichi Japan; 2 Department of Rehabilitation Medicine, School of Medicine, Fujita Health University, 1-98 Dengakugakubo, Kutsukake-cho, Toyoake, 470-1192 Aichi Japan; 3 Faculty of Rehabilitation, School of Health Sciences, Fujita Health University, 1-98 Dengakugakubo, Kutsukake-cho, Toyoake, 470-1192 Aichi Japan; 4 Innovative and Clinical Research Promotion Center, Gifu University Hospital, 1-1 Yanagido, Gifu, 501-1194 Japan

**Keywords:** Stroke, Cerebrovascular disease, Gait independence, Robotics, Rehabilitation, Walking

## Abstract

**Background:**

Robot-assisted gait training (RAGT) is an effective method for treating gait disorders in individuals with stroke. However, no previous studies have demonstrated the effectiveness of RAGT in individuals with acute stroke. This study aimed to investigate the effects of RAGT initiation within 1 week after onset on degree of gait independence in individuals with hemiparetic stroke.

**Methods:**

This retrospective cohort study used propensity-score matching. Individuals admitted to Fujita Health University Hospital after stroke onset and underwent RAGT between March 2017 and June 2023 were enrolled. Ninety-two individuals were eligible and grouped into the acute (≤ 7 days after the onset) and subacute groups (8–90 days after onset). RAGT was conducted using Welwalk, primarily comprising a knee–ankle–foot orthosis type robot worn on one paralyzed lower extremity, with training sessions lasting approximately 40 min/day, occurring 3–7 days/week. The primary outcome was the gait under supervision within 90 days of onset, which was compared between groups using the log-rank test.

**Results:**

After propensity-score matching, 36 individuals were included in the analysis, including 18 each in the acute and subacute groups; the participant demographics were not significantly different between the groups. RAGT was initiated at a median of 6 and 25 days after onset in the acute and subacute groups, respectively. The Kaplan–Meier curves after the log-rank test showed a significantly higher percentage and shorter median days to achieve gait under supervision in the acute group than in the subacute group. The cumulative incidence of gait under supervision events at 90 days after onset was 82.2% and 55.6% in the acute and the subacute groups, respectively. Half of the individuals achieved gait under supervision within 49 days and 75 days in the acute and subacute groups, respectively (*p* = 0.038). No significant differences were observed in the dose of rehabilitation program and gait training per day from onset to achieving gait under supervision.

**Conclusion:**

Initiation of RAGT within 1 week after stroke onset in individuals with hemiparesis may reduce the number of days required to achieve gait under supervision and increase the percentage of gait under supervision.

## Background

Stroke is one of the leading causes of physical disability worldwide, resulting in gait disorders [1, 2]. The percentage of individuals with stroke who have achieved gait independence is estimated to be 41–85% [3]. Gait disorders limit the activities of daily living and social participation [4]. Therefore, improving gait is an important goal in stroke rehabilitation.

Robot–assisted gait training (RAGT) is an effective method for treating gait disorders in individuals with stroke. RAGT can provide intensive, repetitive, and task-oriented training for individuals with hemiplegic stroke who have difficulty walking independently by partially or completely supporting their weight and movement using a robotic control mechanism [5]. A systematic review and meta-analysis reported that RAGT combined with conventional physical therapy for individuals with stroke is effective in improving gait independence within 3 months of stroke onset and in those who are unable to walk [6]. Therefore, RAGT for individuals with stroke is widely recommended in treatment guidelines [7, 8].

A retrospective study reported that early initiation of RAGT improved the degree of gait independence in individuals with subacute strokes, suggesting that early initiation of RAGT may improve the degree of gait independence [9]. It is important to start post-stroke rehabilitation early after stroke onset to achieve favorable clinical outcomes [10]. However, it is not clear how early RAGT should be initiated to improve the degree of gait independence in individuals with stroke who are unable to walk within 3 months of onset. The aforementioned systematic review and meta-analysis included RCTs of individuals with stroke 2–8 weeks after stroke onset [6]. Other systematic reviews investigating the efficacy of RAGT have consistently shown its effectiveness in improving gait ability among individuals with stroke within 3 months after onset but have not included RCTs conducted within the first week after stroke [11–13]. Therefore, the effect of initiating RAGT in the acute phase within 1 week of stroke onset is unclear. We hypothesized that the initiation of RAGT within 1 week may improve the degree of gait independence earlier than initiation of RAGT in the subacute phase. To the best of our knowledge, no previous studies have demonstrated the effectiveness of RAGT in individuals with acute stroke. This study aimed to investigate the effect of RAGT initiation within 1 week after onset on the degree of gait independence in individuals with hemiparetic stroke.

## Methods

### Study design and setting

This study was designed as a retrospective cohort study using propensity-score matching and was conducted at Fujita Health University Hospital. This study was approved by the Fujita Health University Institutional Review Board (HM22–523), and conducted in accordance with the STROBE guideline [14]. The requirement for informed consent was waived due to the retrospective study design, and individuals who did not opt out were included in the final analysis.

### Participants

Participants included individuals who were admitted directly to Fujita Health University Hospital after stroke onset and underwent RAGT between March 2017 and Jun 2023. The enrolled individuals were followed for 90 days after stroke onset, and the follow-up was terminated in August 2023.

The inclusion criteria for RAGT were as follows: (1) hemiparetic stroke within 3 months, (2) risk of knee buckling during gait training using ankle–foot orthosis, (3) permission from the physician in charge to perform RAGT, (4) age 20–85 years, (5) weight 40–95 kg, and (6) height 140–190 cm. The exclusion criteria were as follows: (1) uncontrolled hypertension, (2) tachycardia at rest, (3) training limitations due to cardiac or respiratory dysfunction, (4) lower limb circulatory disorder or peripheral nerve disorder, (5) severe joint contracture or deformity, (6) visual or auditory impairment hindering training, and (7) reaching a score of five or higher in the Functional Independence Measure (FIM) [15, 16] walk scores within 7 days from stroke onset. Individuals who met the inclusion criteria and underwent RAGT were included.

### Rehabilitation

Fujita Health University Hospital has stroke care units and a specialized ward for intensive rehabilitation. All individuals with stroke were admitted to the stroke care units, and rehabilitation was initiated. After acute treatment was completed, the individuals were transferred to the intensive rehabilitation ward. Before transfer to the intensive rehabilitation ward, the rehabilitation program consisted of physical therapy, occupational therapy, or speech–language–hearing therapy for ≤ 180 min/day, 5–7 days/week. After admission to the intensive rehabilitation ward, the rehabilitation program consisted of physical therapy, occupational therapy, or speech–language–hearing therapy for ≤ 180 min/day, 7 days/week. The time allocated for each therapy was coordinated by the rehabilitation physician in charge. RAGT was provided as a part of the physical therapy component of the rehabilitation program. Physical therapy other than RAGT was provided, including range of motion training, muscle strengthening training, and movement training, such as standing and gait training, using a lower limb orthosis. RAGT was conducted for approximately 40 min/day, 3–7 days/week. RAGT was initiated as soon as possible after inclusion and exclusion criteria were met. The criteria for the end of RAGT was mainly when the individual was able to walk overground under supervision. In some cases, RAGT was continued after the individual was able to walk overground under supervision, with the aim of improving the gait pattern. The decision to discharge an individual from the hospital was made by the rehabilitation physician after the individual had maximized their ADL skills during the hospitalization period. The rehabilitation physician made the final decision by adjusting the discharge schedule while adjusting the place of discharge.

### Robotic device

**Fig. 1:**
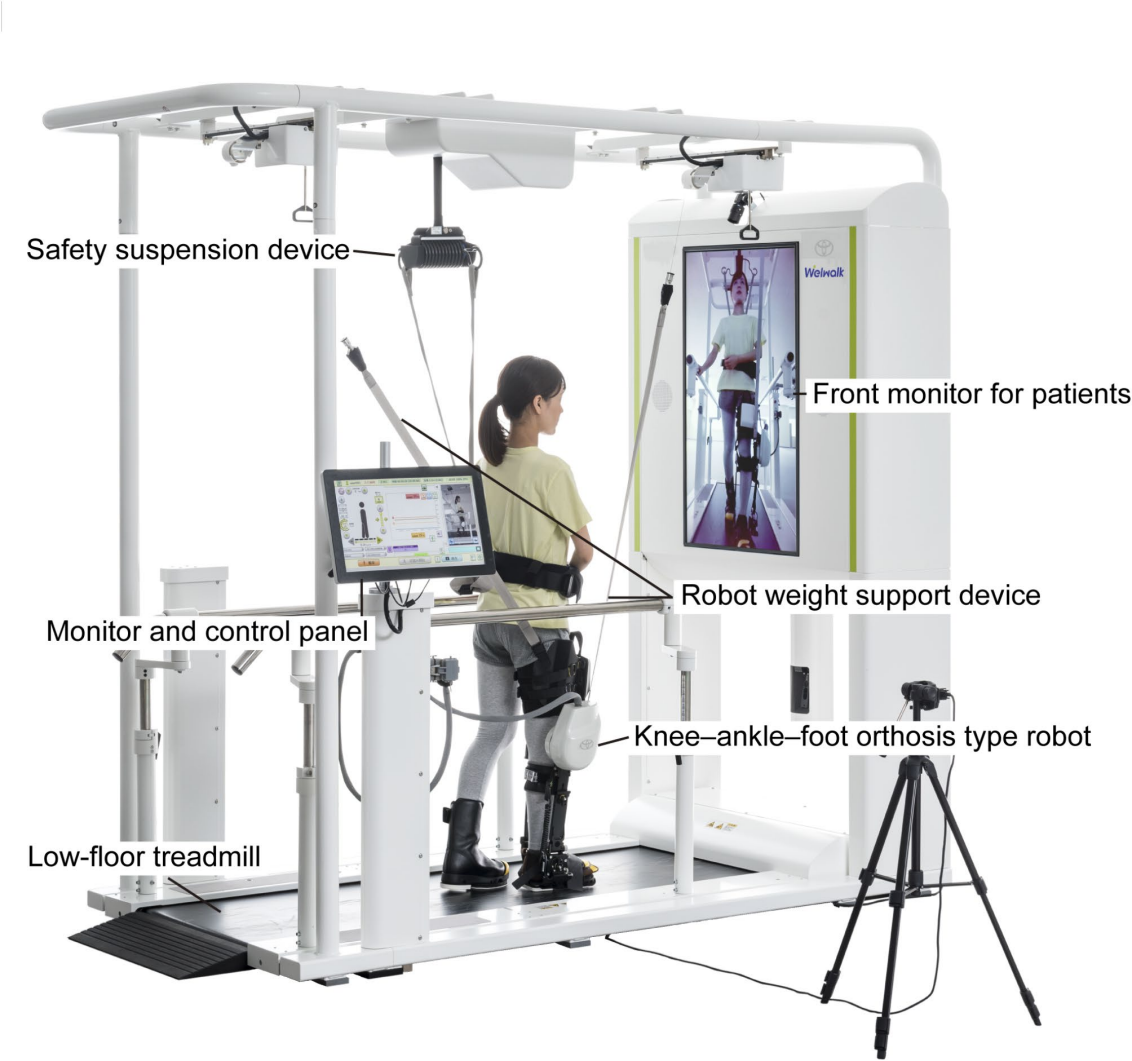
Overview of Welwalk WW–2000

Welwalk (Toyota Motor Corporation, Aichi, Japan; Fig. 1), which was developed to support gait training in individuals with hemiparetic stroke, was used for RAGT. There are two versions of Welwalk: Welwalk-1000 and WW-2000. The device consists of a low–floor treadmill, knee–ankle–foot orthosis type robot, safety suspension device (which can be used for body weight support), robot weight support device, front monitor for patients, and monitor and control panel for the therapist. The knee–ankle–foot orthosis type robot is equipped with a load sensor on the sole, and the gait cycle is detected based on the load. The knee joint motor assists knee joint flexion during the swing phase and knee joint extension during the stance phase. Welwalk-2000 is equipped with a 3D sensor in the front camera and has an additional function to aid gait analysis by detecting abnormal gait pattern. However, the basic structures are almost identical between models, and there is no difference in the function to assist the individual’s gait. In RAGT, the physical therapist sets the degree of robot assistance as low as possible to the extent that it does not seriously disrupt gait pattern or deteriorate compensatory movements. The physical therapist provided minimal support or guidance as needed.

### Outcome measures

The observation period was defined as 90 days from the onset. The primary outcome was the incidence of gait under supervision within 90 days of stroke onset, which was treated as time-to-event data. Gait under supervision was defined as achieving a FIM walk score of 5 or higher. A FIM walk score of 5 or higher, in which gait is physically established, was deemed appropriate for assessing the effects of early RAGT initiation and was thus used as the outcome measure. The FIM consists of 13 motor items and 5 cognitive items and is an index with proven reliability as an indicator for assessing independence in activities of daily living [15, 16]. Each item is scored from one to seven, with 1–4 indicating the need for physical assistance, 5 indicating supervision, 6 indicating modified independence, and 7 indicating complete independence. The FIM motor total score is the sum of the 13 motor items, ranging from 13 to 91 points. The FIM cognitive total score is the sum of the five cognitive items, ranging from 5 to 35 points. The FIM was assessed during the at the start of rehabilitation by the therapist-in-charge.

Information on participant demographics at the start of rehabilitation and interventions was collected. The following participant demographics at the start of rehabilitation were collected: Glasgow Coma Scale (GCS) total score [17, 18], Stroke Impairment Assessment Set (SIAS) motor lower extremity total score [19–21], FIM walk score, FIM motor total score, and FIM cognitive total score.

The GCS has proven to be a reliable indicator of consciousness [17, 18]. The GCS consists of three items: eye opening, verbal response, and motor response, scored from 1 to 4 for eye opening, 1–5 for verbal response, and 1–6 for motor response. The GCS total score is defined as the sum of the eye opening, verbal response, and motor response scores, ranging from 3 to 15, with 3 being the most severe and 15 being normal. The GCS score was assessed at the start of rehabilitation by the therapist-in-charge.

SIAS has proven to be reliable and valid for assessing impairments in individuals with hemiparetic stroke [19–21]. Motor function in the lower extremities, one of the subcategories of this assessment set, consists of hip flexion, knee extension, and foot pad tests. Each item is scored from 0 to 5, with 0 defined as no voluntary joint movement or muscle contraction and 5 as smooth as on the unaffected side. The SIAS motor lower extremity total score is defined as the sum of the hip flexion, knee extension, and foot-pat test scores, ranging from 0 to 15. Each SIAS motor lower extremity item was assessed by the therapist in charge at the start of rehabilitation, and the SIAS motor lower extremity total score was used as an index of the severity of motor paralysis in the lower extremity.

Intervention information was collected from medical records regarding the number of days and time of the rehabilitation program, the time of gait training during the rehabilitation program, and the number of days of RAGT. In addition, the actual gait time (excluding the rest period) and gait distance during RAGT were collected from the Welwalk log data. The following variables were calculated from the collected data: number of days from onset to the start of the rehabilitation program and RAGT, number of days of the rehabilitation program and RAGT from onset to achieving gait under supervision or 90 days after stroke onset, time of rehabilitation and gait training from onset to achieving gait under supervision or 90 days after stroke onset, and actual gait time (excluding rest period) and gait distance during RAGT from onset to achieving gait under supervision or 90 days after stroke onset. Time of rehabilitation and gait training was calculated based on exercise logs recorded in 5-min increments in the medical record.

### Propensity-score matching

We defined the acute phase as duration up to 7 days after stroke onset, referring to the timeline of stroke recovery proposed by Bernhardt et al. [22]. The subacute phase was defined as duration from 8 to 90 days after onset. Individuals were categorized into the acute group if they started RAGT in the acute phase and the subacute group if they started RAGT after the acute phase.

This study performed one-to-one matching with the caliper size set to 0.2 between the acute and subacute groups based on estimated propensity scores for each individual to match the participant demographics at 7 days after stroke onset because individuals in the acute and subacute groups might have had different conditions. Only 6 of the 98 individuals had missing data; even if they were excluded, the results were not considered highly biased. Therefore, individuals with missing data were excluded. Previous studies have reported that factors that predict the gait outcome in individuals with stroke are as follows: age, stroke severity, lower limb motor function, sensory function, trunk function, balance function, cognitive function, visuospatial cognitive function, aphasia, and activity of daily living [23, 24]. Thus, propensity scores were calculated using the following variables: age, affected side, GCS total score, SIAS motor lower extremity total score, FIM motor total score, and FIM cognitive total score, collected as participant demographics at the start of rehabilitation. Previous studies did not report the affected side and the GCS total score as predictors of gait outcome in individuals with hemiparetic stroke [23, 24]. However, we considered that these variables would affect gait outcomes, as the affected side can affect visuospatial cognitive function and the incidence of aphasia [25, 26], and consciousness, as assessed by the GCS, is part of the stroke severity assessment [27, 28]. Therefore, we added these variables as factors to calculate propensity scores.

### Data analysis

In the analysis of the primary outcome, the cumulative incidence of under supervision during the observation period was analyzed using the Kaplan–Meier method, and the log-rank test was used to evaluate the significance of differences in the incidence of gait under supervision between the two groups. The cumulative incidence of gait under supervision events at 90 days after stroke onset and the median number of days from onset to achieving gait under supervision were calculated. Individuals who did not achieve gait under supervision or died during the observation period were censored.

Participant demographics and intervention information were summarized as median and interquartile range for continuous variables and frequency for categorical variables. Differences in these outcomes between the two groups were compared using chi-square and Mann–Whitney U tests. All statistical analyses were performed using EZR (Saitama Medical Center, Jichi Medical University, Saitama, Japan), a graphical user interface for R (R Foundation for Statistical Computing, Vienna, Austria) [29]. Statistical significance was set at values of *p* < 0.05.

## Result

**Fig. 2:**
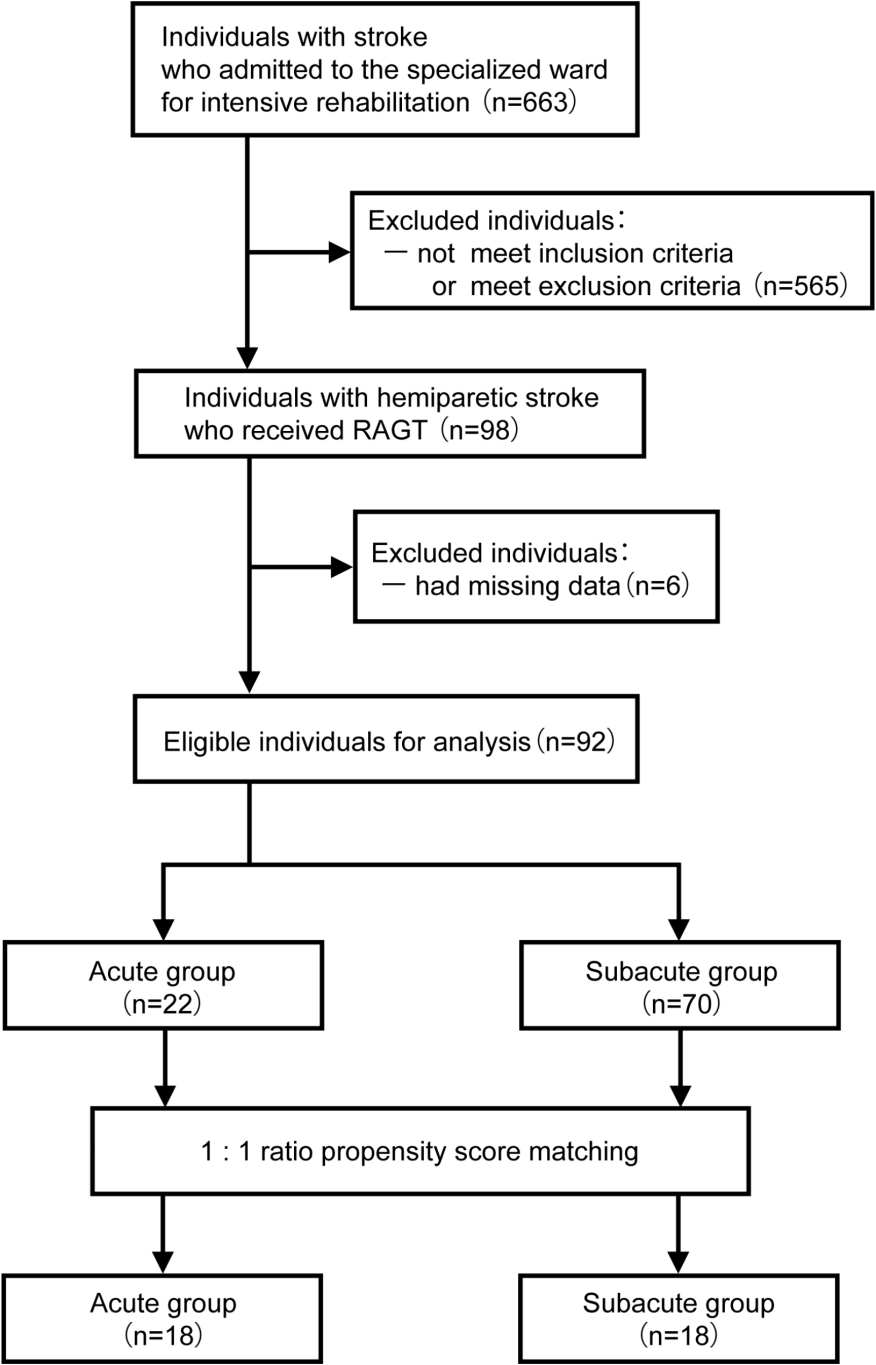
Flow diagram of study participants

During the study period, 663 individuals with stroke were admitted to the emergency units, started rehabilitation, and were transferred to the specialized ward for intensive rehabilitation. Of these, 98 individuals received RAGT. We analyzed the results of these 98 individuals in this study. Of them, 92 were included in the study after 6 with missing data were excluded. The eligible individuals were grouped into the acute group (*n* = 22) and subacute group (*n* = 70). Four individuals in the acute group and 52 in the subacute group were excluded using propensity score matching. Ultimately, 36 individuals were included in the analysis, including 18 each in both groups (Fig. 2).

Participant demographics at the start of rehabilitation in the acute and subacute groups for all individuals and propensity score-matched individuals are shown in Table 1. Before propensity-score matching, the total GCS score was higher in the acute group than in the subacute group. No difference in other variables was observed between the two groups. After propensity-score matching, no significant difference was noted in the participant demographics. RAGT was initiated at a median of 6 days after onset in the acute group and 25 days in the subacute group. The median value of RAGT intervention period was 18.5 days and 18.0 days in acute and subacute groups, respectively.

**Table 1:** Participant demographics

	Unmatched groups			Matched groups		
	Acute group (*n* = 22)	Subacute group (*n* = 70)	*p* value	Acute group (*n* = 18)	Subacute group (*n* = 18)	*p* value
Age, years, median (IQR)	68.5(54.0–76.0)	68.5(56.3–76.0)	0.942	69.0(60.3–76.0)	68.0(55.8–78.0)	0.776
Height, cm, median (IQR)	163.0(155.8–165.0)	162.5(155.0–170.0)	0.734	162.5(155.8–165.0)	160.0(156.3–167.3)	0.975
Weight, kg, median (IQR)	55.6(51.3–69.4)	55.5(50.7–62.0)	0.637	55.6(52.3–69.4)	55.0(45.6–63.4)	0.323
Sex, male / female, n	13 / 9	46 / 24	0.756	10 / 8	14 / 4	0.289
Lesion type, hemorrhage/ infarction / SAH / mixture, n	14 / 8 / 0 / 0	44 / 23 / 2 / 1	0.800	12 / 6 / 0 / 0	12 / 5 / 1 / 0	0.580
Affected side, right / left, n	14 / 8	28 / 42	0.090	10 / 8	11 / 7	0.999
GCS total score, median (IQR)	14.0(14.0–15.0)	13.0(12.0–14.0)	0.006	14.0(13.3–15.0)	14.0(13.0–14.0)	0.359
SIAS motor LE total score, median (IQR)	1.5(0.0–4.8)	1.0(0.0–3.8)	0.523	1.5(0.0–4.0)	1.0(0.0–2.8)	0.602
FIM walk score, median (IQR)	1.0(1.0–1.0)	1.0(1.0–1.0)	0.593	1.0(1.0–1.0)	1.0(1.0–1.0)	0.999
FIM motor total score, median (IQR)	13.0(13.0–14.5)	13.0(13.0–18.0)	0.389	13.0(13.0–16.0)	13.0(13.0–14.5)	0.841
FIM cognitive total score, median (IQR)	20.0(12.3–24.8)	13.5(9.0–22.0)	0.128	18.5(12.3–24.8)	14.5(10.3–21.5)	0.303

FIM, Functional independence measure; GCS, Glasgow Coma Scale; IQR, interquartile range; SAH, subarachnoid hemorrhage; SIAS motor LE, Stroke impairment assessment set motor lower extremity total score

**Fig. 3:**
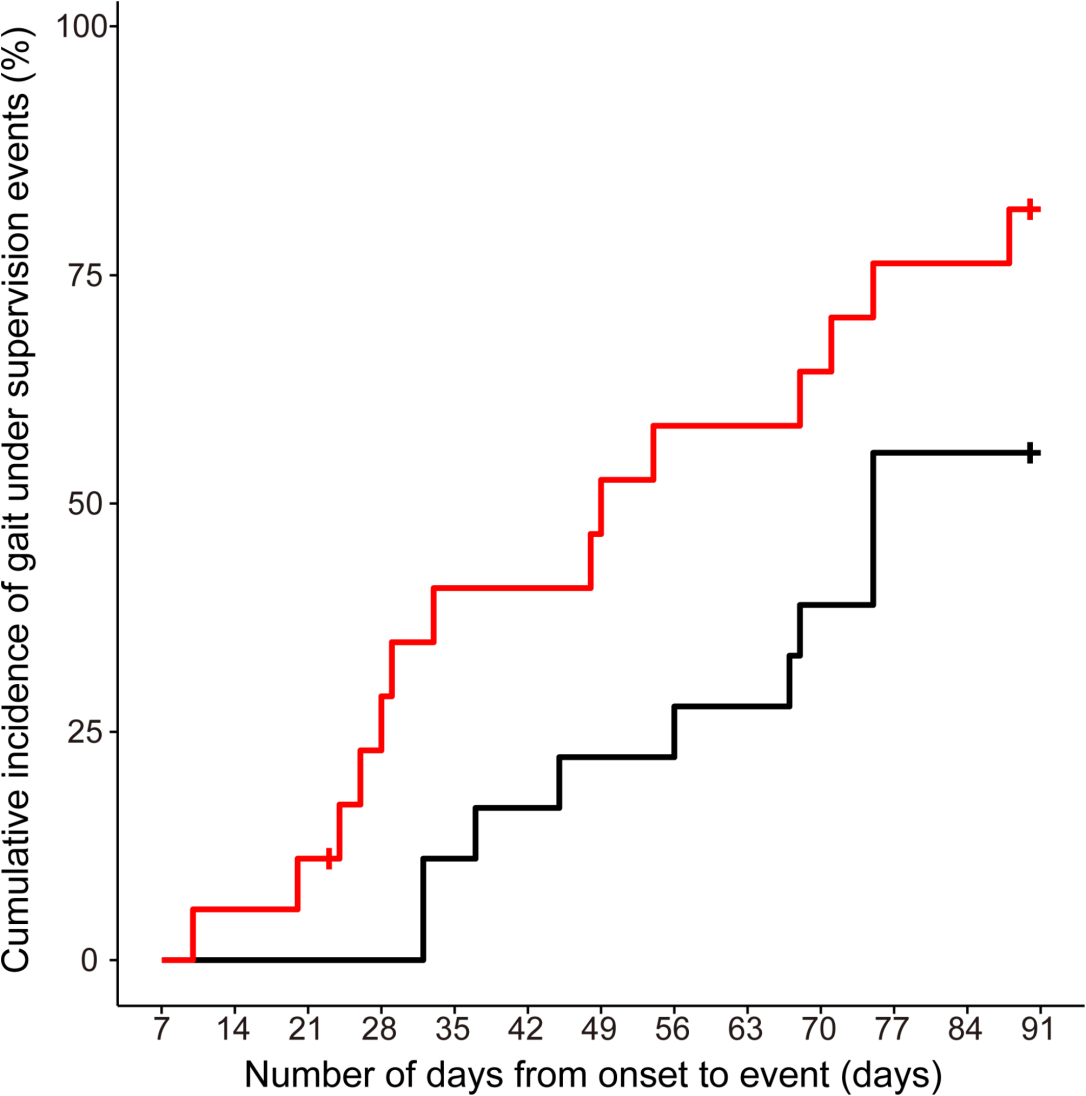
The number and percentage of days from onset to achieving gait under supervision in the acute and subacute groups. The vertical axis shows the cumulative incidence of gait under supervision events, and the horizontal axis shows the number of days from onset to the event. The red line represents data for the acute group, and the black line represents data for the subacute group

The Kaplan–Meier curves after the log-rank test showed a significantly higher percentage and shorter median days to achieve gait under supervision in the acute group than in the subacute group (Fig. 3). The cumulative incidence of gait under supervision events at 90 days after stroke onset was 82.2% (95% confidence interval [CI]: 61.5–95.6%) and 55.6% (95% CI: 34.9–78.4%) for the acute group and the subacute group, respectively. Half of the individuals achieved gait under supervision at 49 days (95% CI: 26.0–75.0 days) and 75 days (95% CI: 56.0–Not calculated days) in the acute and subacute groups, respectively (*p* = 0.038). The number of days from stroke onset to the initiation of RAGT was significantly shorter in the acute group than in the subacute group, but the number of days of RAGT did not differ significantly between the two groups (Table 2). The time of rehabilitation and gait training, actual gait time, and gait distance per day from onset to achieving gait under supervision or 90 days after stroke onset did not differ significantly between the two groups (Table 2).

**Table 2:** Information on intervention

	Acute group (*n* = 18)	Subacute group (*n* = 18)	*p* value
Days from the onset to the initiation of rehabilitation program, median (IQR)	1.0 (1.0–1.0)	1.0 (1.0–1.0)	0.861
Days from the onset to the initiation of RAGT, median (IQR)	6.0 (5.3–7.0)	25.0 (20.5–39.3)	< 0.001
Days of rehabilitation program to event, median (IQR)	42.0 (19.8–62.0)	68.0 (52.8–79.8)	0.030
Days of RAGT to event, median (IQR)	18.5 (14.3–35.8)	18.0 (11.0–27.5)	0.704
Dose of rehabilitation program to event, min, median (IQR)	6,430 (3,430–10,815)	10,960 (8,875–13,050)	0.041
Dose of gait training to event, min, median (IQR)	943 (680–1,855)	1,685 (1,105–2,269)	0.064
Dose of rehabilitation program to event per day, min/day, median (IQR)	170.7 (165.9–176.6)	167.1 (161.9–169.9)	0.248
Dose of gait training to event per day, min/day, median (IQR)	31.9 (26.5–39.7)	28.1 (19.6–34.2)	0.194
Actual gait time during RAGT to event, min, median (IQR)	226.2 (137.4–422.9)	230.4 (130.4–354.6)	0.864
Actual gait time during RAGT to event per day, min/day, median (IQR)	11.7 (8.3–14.2)	12.4 (8.6–14.8)	0.752
Gait distance during RAGT to event, m, median (IQR)	2,426 (1,027–4,033)	2,590 (1,008–3,289)	0.563
Gait distance during RAGT to event per day, m/day, median (IQR)	99.9 (79.1–174.7)	96.9 (81.1–136.2)	0.696

IQR, interquartile range; RAGT, robot–assisted gait training

The length of stay in hospital was a median of 104.5 days (interquartile range [IQR]:77.5–125.3 days) in the acute group and 132.5 days (IQR 114.8–152.3 days) in the subacute group, with the acute group being significantly shorter than the subacute group (*p* = 0.046).

## Discussion

We examined the differences in achieving gait under supervision within 90 days of stroke onset in the acute and subacute groups of demographically similar individuals, categorized whether they started RAGT within 1 week after stroke onset. Our results showed that the Kaplan–Meier curve differed significantly between the two groups. Compared to individuals who did not receive early RAGT, those who received early initiation of RAGT demonstrated exhibited a higher percentage of gait under supervision achieved in a shorter duration. In addition, individuals who started early RAGT were able to be discharged earlier.

A cohort study on the effects of RAGT on the degree of gait independence in individuals with subacute stroke reported greater improvement in the degree of gait independence in individuals who started RAGT approximately 2 weeks after stroke onset than in those who started RAGT approximately 7 weeks after stroke onset [9]. Here, the question has been whether there is any benefit to starting as early as possible, and whether starting within the first week, the acute phase of stroke, is more effective than starting later. The study clearly showed that starting RAGT in the acute phase, i.e. within one week of stroke onset, is more effective than starting it in the later phase.

This study showed that the early initiation of RAGT in the acute phase enhances the degree of gait independence. There are several possible explanations for these results. First, robotic assistance may have contributed to the early provision of task-specific repetitive training and facilitated motor learning. In stroke rehabilitation, intensive and task-specific training, which is an important factor in promoting motor learning, enhances therapeutic effects [30]. However, it is not easy to provide a sufficient amount of task-specific training, such as gait training, to individuals with severe gait impairment in the early post-stroke period because of the high burden on therapist [31–33]. Previous studies have reported that RAGT can reduce the burden on therapists and allow faster speed and longer distance gait training than conventional gait training does [5, 34, 35]. Therefore, we speculate that RAGT can provide repetitive gait training from the early onset of stroke, resulting in early gait under supervision. Second, repetitive gait training early after stroke may prevent disuse. Individuals with stroke are prone to disuse because of their low activity [36]. Individuals with acute stroke are more likely to be inactive than those with subacute stroke; moreover, this tendency is stronger in the early post-onset period [37, 38]. To prevent disuse, it is important to enhance out-of-bed activity during the early stages of stroke [36]. Therefore, early initiation of RAGT may have contributed to preventing disuse that may have occurred during the acute phase, thereby facilitating the early achievement of gait under supervision.

The early initiation of RAGT reduces the number of days to achieve gait under supervision and increases the percentage of individuals achieving gait under supervision. Furthermore, the length of stay in hospital was shorter in the acute group than in the subacute group, and the results suggest that early achievement of gait under supervision through early RAGT may contribute to a shorter length of stay in hospital. This result supports a previous study showing that improvements in independence, including gait, were related to a shorter length of stay in hospital [39]. In addition, there have been reports that reducing the length of hospital stays can lead to a reduction in economic costs, which is meaningful [40]. Furthermore, an increase in the percentage of achieving gait independence may contribute not only to improved activities of daily living but also to a better quality of life [41]. Therefore, we believe that RAGT should be actively implemented soon after the onset of stroke.

This study had several limitations. First, this was a single-center, retrospective cohort study. There are limitations to generalizing the results of this study to all individuals with hemiparetic stroke; thus, caution should be exercised when interpreting these results. Secondly, there may have been a selection bias because the start of RAGT required permission from the physician in charge. Although propensity-score matching was used to match demographic characteristics in the present study, differences in characteristics between groups cannot be removed completely. There may be factors behind the decision of the physician in charge that could not be controlled in this study, including the severity of stroke and presence of comorbidities and neurocognitive disorders such as hemispatial neglect and pusher syndrome. Furthermore, the data used for matching were obtained at the start of rehabilitation and not 7 days after stroke onset. Changes between the start of rehabilitation and 7 days after onset may have led to biased results. Thirdly, we used the FIM walk score as the primary outcome measure. The FIM walk score is an indicator for evaluating the implementation status on the ward, so there is a possibility that it will underestimate the gait ability of the individual. Therefore, it might be better to use an indicator of ability such as the Functional Ambulation Category. In future prospective studies, we will consider using the most appropriate measure of gait ability, including other measures.

## Conclusion

Initiation of RAGT within 1 week after onset in individuals with hemiparetic stroke has the potential to reduce the number of days required to achieve gait under supervision and increase the percentage of individuals who achieve gait under supervision.

## Data Availability

The datasets used and/or analyzed in the current study are available from the corresponding author on reasonable request.

## Abbreviations

FIM: Functional independence measure
GCS: Glasgow coma scale
IQR: Interquartile range
RAGT: Robot–assisted gait training
SIAS: Stroke impairment assessment set
